# Evolving trends in cochlear implant reimplantation: An analysis of causes and outcomes at a tertiary referral cohort

**DOI:** 10.1007/s00405-026-10012-6

**Published:** 2026-02-16

**Authors:** Dong Woo Nam, Yehree Kim, Ye Jun Chun, Hye Rim Park, Jin Hee Han, Ji-Yeon Yang, Min Young Kim, Ngoc-Trinh Tran, Jae Won Yun, Kenneth Lee, Bong Jik Kim, Byung Yoon Choi

**Affiliations:** 1 Department of Otorhinolaryngology, Seoul National University Bundang Hospital, 300 Gumi-Dong, Bundang-Gu, Seongnam, 13620 South Korea; 2 Department of Otorhinolaryngology, Graduate School of Medicine, Chungbuk National University, Cheongju, South Korea; 3 College of Medicine, Korea University, Seoul, South Korea; 4 Department of Otolaryngology-Head and Neck Surgery, University of Texas Southwestern Medical Center, Dallas, TX USA; 5 Department of Otolaryngology-Head and Neck Surgery, Chungnam National University College of Medicine, Daejeon, Republic of Korea; 6 Department of Otorhinolaryngology-Head and Neck Surgery, Seoul National University College of Medicine, Seoul, South Korea; 7 Sensory Organ Research Institute, Seoul National University Medical Research Center, Seoul, South Korea

**Keywords:** Cochlear Implants, Reoperation, Treatment Outcome, Speech Perception

## Abstract

**Purpose:**

This study aimed to characterize evolving causes and outcomes of cochlear implant (CI) revision surgeries in the post-2012 era, a period marked by improved device reliability and changing patient expectations.

**Methods:**

We retrospectively reviewed 1,449 CI procedures performed at a tertiary referral center between 2012 and 2024, identifying 48 revision surgeries. Cases were categorized by implantation site (in-house vs. referral) and classified by revision indication. Device survival was evaluated with Kaplan–Meier and competing risks analyses. Auditory outcomes and electrode positioning were assessed before and after revision.

**Results:**

The in-house revision rate was 1.9%, with a 7-year cumulative device survival of 96.1%. Functional performance concerns (n = 20) emerged as the leading cause of revision, exceeding infection/flap problems (n = 19) and device failure (n = 6). Referral patients more often underwent elective reimplantation for performance optimization, frequently converting from lateral wall to modiolar-hugging arrays. These revisions consistently achieved superior modiolar proximity and significant gains in speech perception, with postoperative imaging confirming successful reinsertion trajectories despite fibrous tracts. Infection remained the predominant early complication, while functional revisions increased gradually over time.

**Conclusion:**

Revision cochlear implantation in the modern era reflects both reduced device failures and the rise of patient-driven revisions for functional optimization. Strategic electrode selection and pursuit of bilateral symmetry can yield meaningful auditory improvements. These findings underscore the evolving role of revision surgery in enhancing CI outcomes, highlighting the importance of individualized decision-making in contemporary practice.

**Supplementary Information:**

The online version contains supplementary material available at 10.1007/s00405-026-10012-6.

## Introduction

Cochlear implant (CI) revision surgery (reimplantation) has historically been driven by factors such as device failure, infection, and electrode displacement or migration [1]. Pre-2012 cohorts reported revision rates up to 7–8%, largely due to major device recalls [1, 2]. However, recent large-scale studies continue to show that device failure remains a leading cause of CI revision (up to 73% of cases in some cohorts), though new complications like flap issues (particularly with diametric magnets) or electrode tip fold-over are also increasingly recognized in newer implant models [2–4].

With advancement in CI technology post-2012, the overall revision rate and the primary causes are expected to have evolved. For the purposes of this analysis, the post-2012 period is considered to follow the major device reliability issues surrounding the 2011 global voluntary recall of the Nucleus CI500 series, which has been attributed by the manufacturer to loss of hermeticity related to variations in the brazing process and microcracks in the braze joint allowing moisture ingress [5]. Consequently, in newer generations of devices, engineering refinements in hermetic sealing, electronics, and magnet design have contributed to a marked reduction in major hard failures, while implanted receiver–stimulators now reach reliability classes that were not attainable in earlier eras.

Beyond improvements in device reliability, the localization of spiral ganglion neuronal tissue in various inner ear malformations has become more precise [6–8]. These advancements have enabled electrode arrays with diverse physical configurations, designed to position channels closer to the spiral ganglion neuronal tissue for more direct stimulation [9]. Consequently, these innovations likely impact both the overall revision rate and the primary reasons for revision in current clinical practice.

Despite technological improvements, infection remains a significant cause for reoperation, underscoring strict surgical technique and postoperative care [10]. Additionally, pediatric implant patients appear to have a higher likelihood of requiring revision surgery, possibly due to earlier implantation age, frequent falls, or other developmental factors [2, 4, 11–13]. This suggests modern CI revision surgery reflects a balance between hardware reliability and patient-specific variables.

While a reduction in device failures is expected to contribute to a lower overall revision rate, the relative proportion of revisions due to factors such as infection or the need for functional optimization may have increased. Indeed, some centers report “elective” or “functional” revisions, wherein a technically functional implant is replaced to enhance electrode placement or speech outcomes [14]. However, comprehensive data on current CI revision surgery, including contemporary rates and shifting causes in the post-2012 era, remain limited [3]. This study examines the rate and primary causes of reimplantation in a cohort of patients who underwent CI at our tertiary referral center after 2012.

## Materials and methods

This retrospective study analyzed data from all CI surgeries performed by a single surgeon at a tertiary referral center, between May 2012 and December 2024. The study period was selected to capture the post-2012 era of advancement in CI technology.

The study population included all patients who underwent CI surgery during this period. Patients requiring revision surgery (reimplantation) were identified and categorized into two groups: (1) in-house revisions, where the initial CI implantation was performed at our hospital, and (2) referral revisions, where the patient had received their initial CI at another institution before presenting to our hospital for revision.

Data collected included patient demographics, details of the initial CI surgery and any subsequent revisions, and the reasons for revision. Reasons for revision were classified as: (1) device failure, (2) infection/flap problems, (3) electrode migration, (4) magnet weakening, and (5) functional performance concerns. The "functional performance concerns" category encompassed cases where revision was pursued despite the existing device functioning without evidence of failure, infection, or migration. These cases were further classified into three subcategories for clarity: (a) soft failure, (b) obligatory functional revision (revision due to absent or minimal functional benefit despite a normally functioning electrode), and (c) optional functional revision (revision for improved performance despite demonstrable functional benefit from the existing electrode). In this study, soft failure was defined in accordance with the 2005 Consensus Statement [15] as a working diagnosis in which integrity testing of the internal device remained within normal limits, yet the patient exhibited an unexplained decrement in CI performance or aversive non-auditory symptoms after exclusion of medical, surgical, and programming causes. Optional functional revisions were mainly motivated by appreciation of a partially inserted electrode or a desire to match or surpass the performance of a better-performing contralateral CI. To evaluate the effectiveness of revision surgery for patients in the "functional performance concerns" category, sentence scores and Categories of Auditory Performance (CAP) were compared before and after revision surgery. These analyses were performed separately for each subcategory using the Wilcoxon signed-rank test.

To compare the distribution of reasons for revision between in-house and referral cases, Fisher's exact test was performed.

For the device survival analysis, only in-house CI ears implanted between 2012 and 2022 with a minimum follow-up of two years were included to ensure adequate data maturity. Device survival was defined as the time from implantation to device explantation for any reason. Patients who did not experience device explantation were censored at their last follow-up date. Device survival was analyzed using the Kaplan–Meier method.

To investigate the cumulative incidence of revision surgery due to different causes, we performed a competing risks analysis using the Fine-Gray method for the cause-specific cumulative incidence function (CIF).

In a subset of patients, modiolar proximity was assessed postoperatively via plain X-ray using a previously described method to estimate the electrode’s distance from the modiolus [16]. This technique uses a standard plain X-ray (transorbital or modified Stenver’s view) to evaluate the relative position of the electrode array to the central axis of the cochlea, offering a practical approach for assessing modiolar proximity.

For patients with bilateral hearing, sentence scores and CAP were calculated for each ear individually, with the contralateral hearing aid or cochlear implant turned off.

All statistical analyses were conducted using R version 4.5.0. A p-value of < 0.05 was considered statistically significant.

This retrospective study was conducted in accordance with the Declaration of Helsinki and was approved by the Institutional Review Board (IRB) of the participating institution (IRB approval number: B-2503–960-101), which granted a waiver of the requirement for informed consent.

## Results

A total of 48 CI reimplantation surgeries were performed. Twenty-seven of these patients had their primary surgery at our hospital (in-house revisions), while 21 were for patients who received their primary CI elsewhere (referral revisions). Among the 1,401 ears that underwent their first CI at our center, 27 required revision surgery, representing an in-house revision rate of 1.93% (Table 1). The revision cohort encompassed 48 revision cases involving both pediatric and adult CI recipients (mean age at revision: 21.5 years; range: 1.1–77 years). Notably, the age distribution varied by indication reflecting the underlying etiology. The 'obligatory functional revision' group (mean age: 20.4 years; range: 3–63 years) included both early revisions for pediatric complications and delayed revisions in adults with long-standing congenital anomalies. In contrast, the 'optional functional revision' group (mean age: 28.0 years; range: 6–40 years) consisted largely of patients capable of subjective performance comparison.

**Table 1:** Cochlear implant device type and in-house revision rates

	**Primary Surgery**	**In-house revision**	**In-house Revision Rate (%)**
Cochlear			
- SME	1197	17	1.42
- 24RE-ST	23	2	8.70
- 24RE-CA	25	2	8.00
- SSE	74	1	1.35
Med-EL SSE	82	5	6.10
All	1401	27	1.93

Abbreviation: SME, slim modiolar electrode; 24RE-ST, full-band straight electrode; 24RE-CA, contour-advance; SSE, slim straight electrode; NA, not applicable

Analysis of revision causes between in-house and referral cases revealed that, notably, the most prevalent indication for reimplantation was functional performance concerns (n = 20), rather than traditional causes. The remaining revisions were attributed to infection/flap problems (n = 19), device failure (n = 6), electrode migration (n = 2), and magnet weakening (n = 1) (Table 2).

**Table 2:** Causes of cochlear reimplantation: In-house vs Referral

	**Device** **failure**	**Electrode** **migration**	**Magnet** **weakening**	**Infection/flap problems**		**Functional performance concerns**			**All**
				**Infection**	**Epidural hematoma**	**Soft** **Failure**	**Obligatory** **functional**	**Optional** **functional**	
In-house	4	2	1	16	1	1	0	2	27
Referral	2	0	0	2	0	3	9	5	21
All	6	2	1	18	1	4	9	7	48

A statistically significant difference was observed in the distribution of the five revision causes when comparing in-house with referral cases (Fisher's exact test, p < 0.001). Referral cases more frequently involved reimplantation for functional performance optimization, whereas in-house cases showed a higher proportion of revisions due to infection/flap problems.

**Fig. 1:**
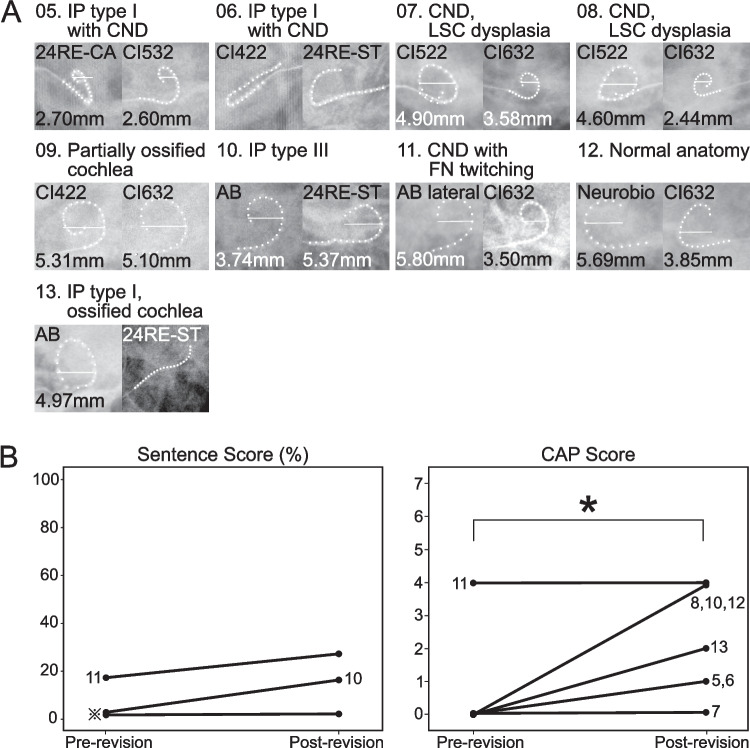
Transorbital views and auditory outcomes of obligatory functional cochlear reimplantation cases. (A) Transorbital views before and after surgery for obligatory functional revision cases are presented. In each case, the left side shows the preoperative view, and the right side shows the postoperative view. Types of cochlear anomalies, device types, and electrode diameters are indicated. For clarity, dots representing each electrode channel are emphasized. (B) Auditory outcomes after obligatory functional cochlear reimplantation, shown by sentence scores (%) and Categories of Auditory Performance (CAP) scores before and after revision surgery. Cases marked with ※ indicate patients who maintained a sentence score of 0% both before and after reimplantation (Pt 5, 6, 7, 8, 12, 13). * Abbreviation: HF, AB HiFocus

**Fig. 2:**
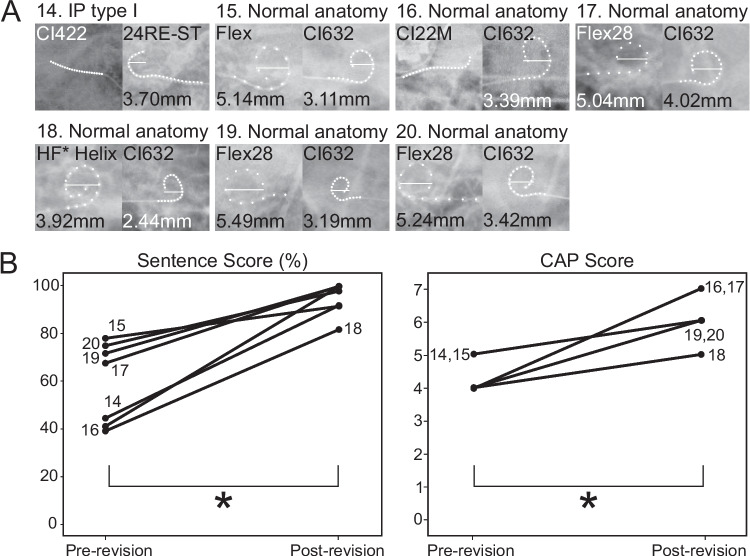
Transorbital views and auditory outcomes of optional functional cochlear reimplantation cases. (A) Transorbital views before and after surgery for optional functional revision cases are shown. In each case, the left side is the preoperative view, and the right side is the postoperative view. Cochlear anomaly types, device types, and electrode diameters are specified. Electrode channel dots have been emphasized to enhance readability. (B) Auditory outcomes after optional functional cochlear reimplantation, demonstrated by sentence scores (%) and Categories of Auditory Performance (CAP) scores before and after revision surgery. * Abbreviation: HF, AB HiFocus

The 20 performance-related functional revisions were classified into three categories: (Table 2, Table 3, Figs. 1 and 2).

**Table 3:** Patient profiles undergoing cochlear reimplantation due to functional performance concerns

ID	Sex	Ear	Cochlear Anomaly	Age at revision	Group	Pre-revision Device	Post-revision Device	Pre-revision Audio	Post-revision Audio
Soft failure									
1	F	Lt	Normal anatomy	57	in-house	Med-EL (Flex28)	Med-EL (Flex28)	2/4	0/4
2	M	Lt	CND	24	referral	AB HiFocus	CI532	34/4	40/4
3	M	Rt	Normal anatomy	23	referral	AB HiFocus	CI532	90/5	100/7
4	M	Rt	Normal anatomy	28	referral	AB HiFocus 1j	CI632	0/1	56/4
Obligatory functional									
5	M	Rt	IP type I with CND	4	referral	24RE-CA	CI532	0/0	0/1
6	M	Lt	IP type I, CND with CSF leak	4	referral	CI422	24RE-ST	0/0	0/1
7	M	Rt	CND, LSC dysplasia	3	referral	CI522	CI632	0/0	0/0
8	M	Lt	CND, LSC dysplasia	3	referral	CI522	CI632	0/0	0/4
9	M	Rt	Partially ossified cochlea	48	referral	CI422	CI632	NA	NA
10	M	Rt	IP type III	19	referral	AB HiFocus Helix	24RE-ST	0/0	14/4
11	F	Rt	CND with FN twitching	17	referral	AB HiFocus 1j	CI632	16/4	26/4
12	M	Lt	Normal anatomy	63	referral	Neurobiosis	CI632	0/0	0/4
13	M	Rt	IP type I, ossified cochlea	23	referral	AB HiFocus 1j	24RE-ST	0/0	0/2
Optional functional									
14	M	Lt	IP type I	14	referral	CI422	24RE-ST	45/5	92/6
15	M	Rt	Normal anatomy (*OTOF* variant)	6	referral	Med-EL (Flex)	CI632	80/5	92/6
16	F	Rt	Normal anatomy	40	referral	CI22M	CI632	42/4	100/7
17	F	Rt	Normal anatomy (*LOXHD1* variant)	37	in-house	Med-EL (Flex28)	CI632	68/4	100/7
18	M	Rt	Normal anatomy (*GJB2* variant)	26	referral	AB HiFocus Helix	CI632	40/4	82/5
19	M	Lt	Normal anatomy (*PRPS1* variant)	37	referral	Med-EL (Flex28)	CI632	72/4	100/6
20	F	Lt	Normal anatomy (*CDH23* variant)	36	in-house	Med-EL (Flex28)	CI632	76/4	98/6

^*^ Pre- and post-revision audio performance was reported using sentence/CAP scores from speech evaluations

1. Soft failure (n = 4): These cases required frequent check-ups due to intermittent device malfunctions or unexplained performance decrements, despite normal integrity test results and the absence of identifiable medical, surgical, or programming causes.
2. Obligatory functional revision (n = 9): These cases involved limited functional benefit, including no sound detection, critical facial nerve (FN) twitching, or no open-set speech recognition, despite confirmed electrode functionality. Although the intracochlear trajectories of the electrode arrays used in the initial implantation and reimplantation were markedly different in most cases, we were able to successfully insert the new electrode array along the intended trajectory (Fig. 1A).

Among the nine cases, three (Pt 8,10 and 12) showed an increase of at least four points on their CAP scores **(**Fig 1B**)**. In two additional cases (Pt 5, 6), which initially exhibited no sound detection, patients began detecting sounds under audio-only conditions **(**Fig. 1B**)**. Patient 11 with cochlear nerve deficiency (CND) was unable to use the implant due to FN twitching; after reimplantation, the twitching diminished, and sentence comprehension scores improved **(**Fig. 1B**)**. Another patient 13 presented with a severely ossified cochlea; during reimplantation, we discovered the original electrode was placed extra cochlearly and inserted a new electrode into the remaining lumen, enabling the patient to respond when called by name **(**Fig. 1B**)**. Statistical analysis revealed no significant change in sentence scores (p = 0.371), whereas CAP scores showed a statistically significant improvement following surgery (p = 0.034).

3. Optional functional revision (n = 7): Most of these patients received their implants in stages, often many years apart. This type of revision typically arose in cases where CI was performed sequentially in both ears using different electrode arrays (e.g., a slim lateral wall type vs. a modiolar-hugging type). Patients who opted for this reimplantation demonstrated a clear preference for one electrode design, finding the functional outcome of the non-preferred side significantly inferior. In all such cases, patients consistently favored the electrode type used in the second implantation. Consequently, they elected to replace the electrode in the initially implanted ear with one matching the design of the contralateral side. Notably, in every instance, the electrode in the first-implanted ear was a slim lateral array, whereas the second-implanted ear received a slim, modiolar-hugging array (SME).

In all cases requiring conversion from lateral wall electrodes to SMEs, the insertion was accomplished successfully, with post-revision imaging **(**Fig. 1A, 2A**)**, suggesting that pre-existing fibrous tracts from previous implantations did not significantly impede the positioning of SMEs closer to the modiolus as intended (Table 3).

In all cases, superior modiolar proximity was successfully achieved in revision cases **(**Fig. 2A**)**. Patient 14 is an exception: due to an incomplete partition (IP) Type I malformation, reimplantation was performed to attempt full insertion of an electrode array that was previously could only be partially implanted. Notably, the discomfort that had compelled this patient to undergo reimplantation—at their own expense and time—on a side that was otherwise functioning reasonably well was entirely alleviated following the functional optional revision. This was true for all cases as similar results were subjectively reported by all individuals. In line with this, post-revision assessment of the reimplanted side demonstrated significant improvement in word/sentence discrimination in all optional functional revision cases **(**Fig. 2B**)**. Wilcoxon signed-rank tests demonstrated statistically significant improvement in both sentence scores and CAP scores following revision (p = 0.031, p = 0.035, respectively). Notably, five of these seven individuals had known genetic variants linked to favorable CI outcomes.

**Fig. 3:**
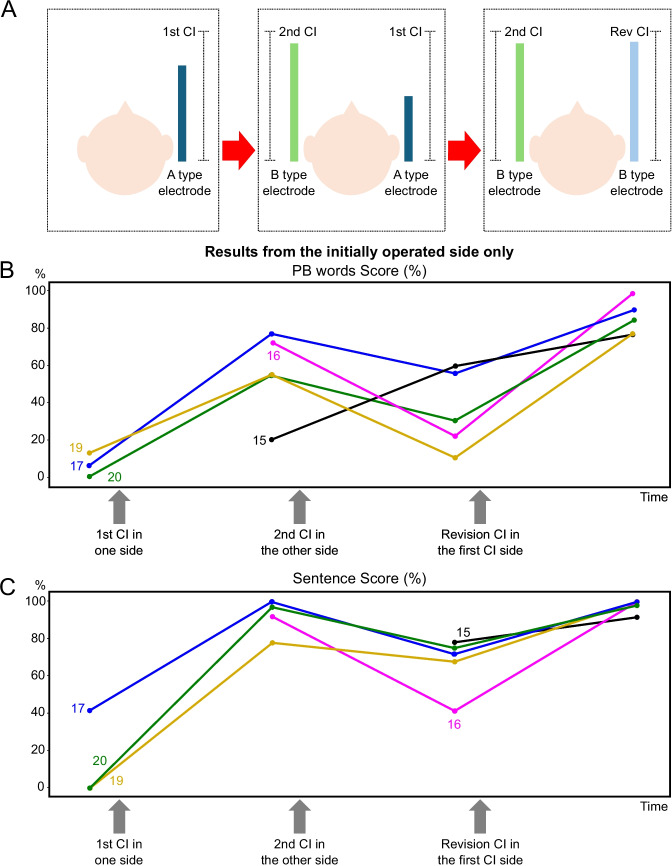
Interaural performance changes in sequential cochlear implantation and first-ear reimplantation cases. (A) Schematic illustration depicting electrode type and implantation sequence in patients undergoing sequential cochlear implantation followed by First-Ear Reimplantation. Initially, patients received a first CI with an A-type electrode. Subsequently, a second CI with a B-type electrode was implanted in the contralateral ear, leading to interaural asymmetry in auditory outcomes. This asymmetry prompted revision surgery of the first CI, replacing the A-type electrode with a B-type electrode to achieve bilateral symmetry. (B) Changes in phonetically balanced words scores (%) with first CI alone across the three stages: initial first CI (1st CI), second CI (2nd CI), and reimplanted first CI (Rev. 1 st CI). Each colored line represents an individual patient (Patients 15, 16, 17, 19, and 20), with patient IDs indicated. (C) Changes in sentence scores (%) with first CI alone across the same three stages. Similar to panel B, each line traces the sentence score trajectory for each patient. Both panels demonstrate that auditory performance, initially asymmetrical following sequential implantation with different electrode types, improved after reimplantation of the first CI with an electrode matching that of the second CI

Among these optional functional revision cases, five patients had undergone “first-ear reimplantation after sequential CI”. In most of these patients, the interaural performance gap had widened significantly prior to revision, with the side targeted for revision often outperforming the original unilateral performance levels (Fig. 2 and 3). This observation suggests that substantial asymmetry in functional outcomes between bilateral implants can prompt patients to seek revision even if the earlier-implanted side had previously provided acceptable performance as a stand-alone implant.

These functional revisions occurred despite comprehensive testing showing no evidence of device malfunction as evidenced by integrity check reports confirming normal electrode function (see Online Resource 1, which shows the integrity test results).

Among these cases, several involved a deliberate change to a different type of electrode based on the underlying rationale. First, in most obligatory functional revision cases where improved neuronal proximity was an objective (Pt 5, 7, 8, and 11), SMEs were deliberately chosen to achieve closer positioning to the modiolus, the anatomical region densely populated by spiral ganglion neurons [9]. In cases of cochlear malformation with an absent modiolus (Pt 10), we selected a full-band lateral wall electrode to maximize cochlear coverage of potentially sparse neural elements and to minimize insertion trauma and electrode misplacement. This strategy was particularly useful in patients with CND and IP type III, an X-linked inner-ear anomaly characterized by absence of the modiolus and of the bony partition between the basal turn of the cochlea and a bulbous internal auditory canal, resulting in an abnormally wide communication between the cochlear base and the internal auditory canal and a high risk of cerebrospinal fluid gusher and electrode misplacement into the internal auditory canal during cochlear implantation[17, 18]. Second, where deeper or full insertion was the goal, electrode replacement allowed more complete coverage of the cochlear duct. For instance, patient 14 with IP type I malformation or partially inserted electrodes in the initial surgery underwent reimplantation to achieve full insertion with straight electrodes. Finally, for patients with ossified cochleae or complex inner ear malformations, flexible or thin electrodes were used to navigate the limited space more effectively. These targeted approaches demonstrate that electrode selection in revision surgery is not merely a technical adjustment but a strategic decision grounded in anatomical and functional considerations.

For the device survival analysis of 885 in-house CI ears (mean follow-up 4.8 years), 23 (2.6%) required reimplantation. The Kaplan–Meier analysis demonstrated a cumulative survival probability of 96.1% at 7.1 years post-implantation, and a device survival probability (due to hard failure) of 99.1% at 6.3 years, indicating high long-term device reliability. (see Online Resource 2A, which shows Kaplan–Meier for device survival).

The competing risks analysis for reimplantation causes revealed that infection/flap-related revisions typically occurred in the early postoperative period, while functional performance-driven revisions showed a more gradual increase over time. (see Online Resource 2B, which shows competing risk analyses for reimplantation causes).

## Discussion

This study presents a comprehensive 12-year analysis of CI revision surgeries, revealing a high cumulative survival rate of 96.1% at seven years, reflecting advancements in CI technology.

This observation is consistent with other large-scale studies [4]. This underscores the success of engineering efforts to minimize device failures, though it remains a significant concern, particularly in pediatric populations [19]. Despite these improvements, our data demonstrate that infection remains a primary cause of reimplantation, highlighting the continued importance of stringent infection prevention protocols. Notably, while flap-related complications have been documented elsewhere [3], we did not encounter any cases of speech processor retention failure in our cohort, which we attribute, in part, to our technique of placing the coil at the lateral side of the temporalis muscle layer for patients with thick scalp [20].

An important methodological strength of our study is the use of CIF analysis. Unlike conventional Kaplan–Meier analysis, CIF analysis accounts for competing risks and provides a more accurate depiction of how each cause of revision accumulates over time. This approach clarifies how an early event (e.g., infection) can prevent the occurrence of a later event, thus giving clinicians a more nuanced view of when and why revisions happen.

Our cohort's composition, predominantly consisting of one manufacturer's SMEs (CI532 and CI632), provides unique insights into device-specific outcomes. This homogeneity allows for a more focused analysis of these specific electrode types' performance characteristics. For instance, we observed only one case of electrode migration, which occurred in a patient with a CI422 electrode, while no such complications were noted in patients with SMEs, suggesting that electrode design might play a crucial role in complication profiles.

Interestingly, we observed distinct differences in the motivations for revision between in-house and referral cases. While in-house revisions more frequently addressed conventional indications, referral revisions frequently focused on optimizing functional performance. These differences likely reflect referral patterns—patients with device complications typically return to their original surgical center, whereas those seeking performance optimization tend to be referred to tertiary centers. This trend aligns with emerging literature noting that up to 21% of revision surgeries involve switching from one manufacturer to another, without negative impacts on outcomes [21]. This finding emphasizes patient-centered care, as some patients seek further improvements despite having a functioning CI. However, as noted in recent literature, there is no broad consensus yet on precise indications or best practices for elective technical upgrades, pointing to a growing area of clinical debate [19].

Our analysis furthers understanding of "optional functional revisions," which may occur when subtle performance discrepancies exist between bilateral implants. These revisions often lead to significant improvements, suggesting a technically functional implant may be suboptimal if electrode positioning or design is mismatched to patient needs. Importantly, our findings demonstrate that conversion from lateral wall electrodes to modiolar hugging electrodes is technically feasible, with radiographic evidence confirming successful achievement of better modiolar proximity. This suggests that pre-existing fibrous tracts from previous implantations do not necessarily prevent the establishment of new, more medial electrode trajectories. In cases of functional optional revision, the observed improvement may be attributed to the use of identical electrode arrays bilaterally, enhancing auditory balance or to the revised electrode achieving closer proximity to key neural structures. From a practical standpoint, we have adopted a conservative, stepwise approach before offering optional functional revision: first, we attempt optimized re-mapping of the existing device and focused auditory rehabilitation to maximize performance over at least one year; second, we confirm on ear-specific testing that, despite these efforts, the earlier-implanted ear remains clearly inferior in subjective and objective performance compared with the contralateral CI; and, finally, we consider whether this persistent asymmetry substantially compromises overall patient satisfaction and communication in daily life. Only when all of these conditions are fulfilled do we regard "functional optional revision" as a reasonable indication for reimplantation.

In certain cases, patients initially fitted with a slim straight electrode (SSE) who later received a contralateral SME noticed performance differences, leading them to consider revision of the original device. While research suggests that SME may provide better modiolar proximity and reasonable preservation of residual hearing [22–25], we emphasize that the motivation for such revisions does not necessarily stem from an inherent superiority of one design but rather from the patients’ desire for more consistent bilateral input. Thorough counseling remains essential to ensure patients understand the potential benefits against the surgical risks.

Based on our findings, in cases where sequential CI recipients experience pronounced asymmetry, patients often express significant dissatisfaction with the initial device and advocate strongly for reimplantation toward bilateral device type symmetry. It is noteworthy that those who perceive a difference significant enough to seek functional reimplantation consistently choose the later-implanted side, which in our cohort was always an electrode with better modiolar proximity.

A particularly notable observation was the marked improvement in open-set speech recognition among patients who underwent "obligatory functional" revisions aimed at optimizing electrode placement. Patients revised from an SSE to an SME, or from a modiolar-hugging array to a full-band electrode for IP type III, demonstrated considerable gains. These improvements likely stem from achieving closer proximity to the cochlear neural elements, thereby enhancing the efficiency of neural stimulation. The technical success of these trajectory conversions suggests that fibrous tissue formation from previous implantations does not necessarily prevent establishing new electrode pathways closer to the modiolus [21]. Likewise, in soft failure cases, the decision to reimplant was made as a diagnosis of exclusion and as a working diagnosis, and the subsequent improvement in auditory performance after reimplantation supports the clinical validity of this categorization in the contemporary CI era.

Our findings regarding pediatric patients deserve attention, as several studies have documented that this population demonstrates a higher tendency toward reoperation [4, 26]. This elevated risk in children is clinically significant, underscoring the importance of close follow-up and robust counseling for families.

Additionally, while other studies have reported flap-related issues as a growing cause of revision surgery, particularly with the advent of diametric magnets [3], we did not encounter any cases of retention failure of the speech processor in our cohort. This discrepancy may be attributed to several factors, including differences in surgical technique, patient populations, or postoperative care protocols between our center and those described in other studies [20]. Placing the coil more laterally in patients with thicker scalps may be a critical factor in preventing retention issues.

This study has some limitations. Its retrospective, single-surgeon design may limit generalizability. While our study population is unbalanced with respect to CI device manufacturers, this characteristic strengthens our ability to draw focused conclusions about these electrode type's performance. Looking forward, further psychoacoustic studies examining patients with bilateral implants using different electrode types could help better characterize subjective auditory differences and guide future revision indications.

Despite these limitations, our study provides valuable contemporary data on CI revision. The high number of referral reimplantation cases in our cohort is partly explained by the Korean healthcare system, where patients can visit any institution, and our center often serves as a last resort. The high device survival rate reinforces the success of CI technology, yet the persistent risk of infection necessitates continued surgical vigilance. Furthermore, the increasing focus on functional optimization underscores the need for proactive identification of patients who may benefit from revision.

In summary, this twelve-year analysis reveals that while overall device survival is high, infection persists as a leading cause of revision, and device failure rates have diminished significantly post-2012. Our findings also highlight that choosing an appropriate electrode—such as an SME for certain CND cases or a full-band straight electrode for IP type III anomalies—can optimize cochlear-neural proximity. Additionally, revisions undertaken for functional optimization, including efforts to unify electrode types or achieve better cochlear placement, can enhance auditory outcomes. Finally, the study underscores the critical role of revision surgeries in improving long-term hearing results for CI recipients, affirming that patient-centered decision-making and careful surgical planning remain paramount for successful outcomes.

## Supplementary Information

Below is the link to the electronic supplementary material.

Supplementary file1 (PDF 106 KB)

Supplementary file2 (PDF 176 KB)

## Data Availability

The datasets generated and/or analyzed during the current study are available from the corresponding author on reasonable request.
