# Supplementary material for: Evolving trends in cochlear implant reimplantation: An analysis of causes and outcomes at a tertiary referral cohort
Source: Eur Arch Otorhinolaryngol. 2026 Feb 16;283(5):2927–37. doi: 10.1007/s00405-026-10012-6 (PMC13152954; doi:10.1007/s00405-026-10012-6)
Supplement: Supplementary file 1 — Supplementary file1 (PDF 106 KB) [file 405_2026_10012_MOESM1_ESM.pdf]

Online Resource 1. The integrity test results of Pt 3 (from the soft failure category of functional revision) and Pt 18 (from the optional functional revision category) are provided. These tests do not show any detectable abnormality

Patient 3: Soft failure

Operation Performed	Test Results
**External Visual Inspection** - To identify anomalies or damage associated with the failure mode<br>**Comments**: This inspection revealed the electrode was severed. This is believed to have occurred during revision surgery.	N/A
**X-Ray Inspection** - To Identify broken wires and loose components associated with the failure mode.<br>**Comments**:	Pass
**Lock Test** - To assess the ability to connect and maintain communication between the Implant and Sound Processor.<br>**Comments**:	Pass
**Wet Impedance Test** - To measure electrode channel impedances in saline.<br>**Comments**: Unable to measure impedances due to the electrode condition.	N/A
**Output Capacitor Leakage Test** - To detect excess DC leakage across the electrode output blocking capacitors.<br>**Comments**:	Pass

Analysis Summary:

This device was explanted for medical reasons. The device passed the tests performed. This older device configuration is not currently manufactured.

Primary Reason: Medical Explant

Secondary Reason: Elective Removal - Technology Upgrade

Patient 18: Optional functional revision

Operation Performed	Test Results
**External Visual Inspection** - To identify anomalies or damage associated with the failure mode.<br>**Comments**: This inspection revealed exposed electrode wires. This is believed to have occurred during revision surgery.	N/A
**X-Ray Inspection** - To Identify broken wires and loose components associated with the failure mode.<br>**Comments**: This inspection revealed broken electrode wires. This is believed to have occurred during revision surgery.	N/A
**Lock Test** - To assess the ability to connect and maintain communication between the Implant and Sound Processor.<br>**Comments**:	Pass
**Wet Impedance Test** - To measure electrode channel impedances in saline.<br>**Comments**: Impedance values of channels 8, 11 and 13 were out of range. This is due to the electrode condition.	N/A
**Output Capacitor Leakage Test** - To detect excess DC leakage across the electrode output blocking capacitors.<br>**Comments**:	Pass

Analysis Summary:

This device was explanted for medical reasons. The device passed the tests performed. This older device configuration is not currently manufactured.

Primary Reason: Medical Explant

Secondary Reason: Elective Removal - Technology Upgrade
