# Supplementary material for: Evolving trends in cochlear implant reimplantation: An analysis of causes and outcomes at a tertiary referral cohort
Source: Eur Arch Otorhinolaryngol. 2026 Feb 16;283(5):2927–37. doi: 10.1007/s00405-026-10012-6 (PMC13152954; doi:10.1007/s00405-026-10012-6)

Online Resource 2. Device survival and causes of cochlear reimplantation over time. (A) Kaplan-Meier survival curve for cochlear implant device survival. The analysis includes 885 CI ears implanted in-house between 2012 and 2022, with a minimum follow-up of two years. The cumulative survival probability was 96.1% at 7.1 years post-implantation. (B) Cumulative incidence function curves for cochlear reimplantation causes, analyzed using a competing risk model. The analysis categorizes reimplantation reasons into seven distinct causes, illustrating their respective cumulative incidence over time. Infection was the most common cause of reimplantation, occurring at the highest rate, particularly in the early postoperative period.

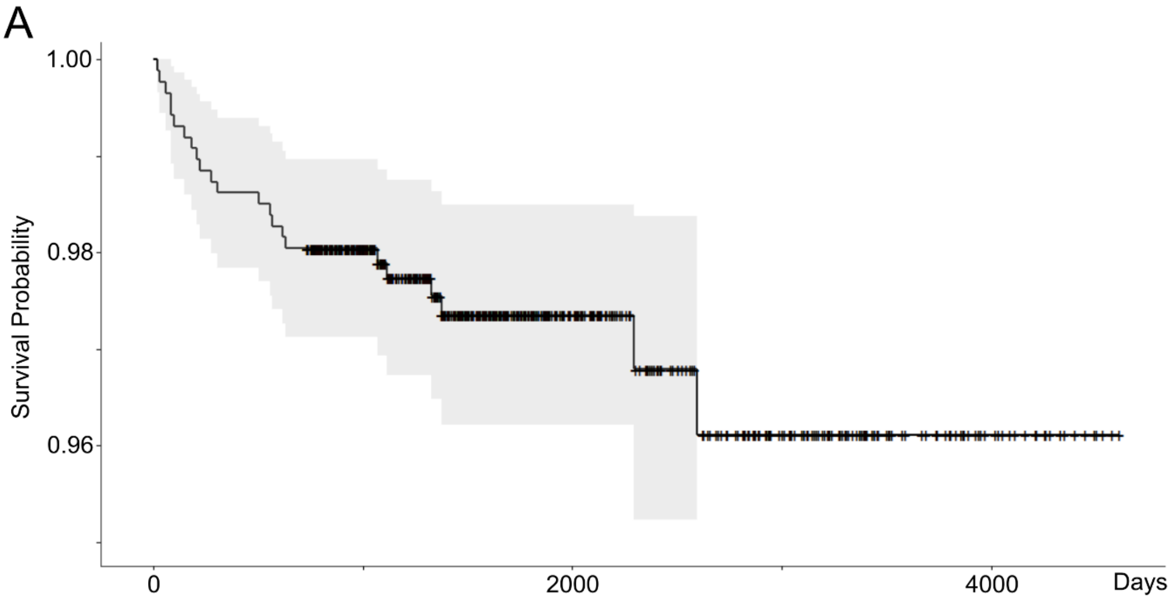

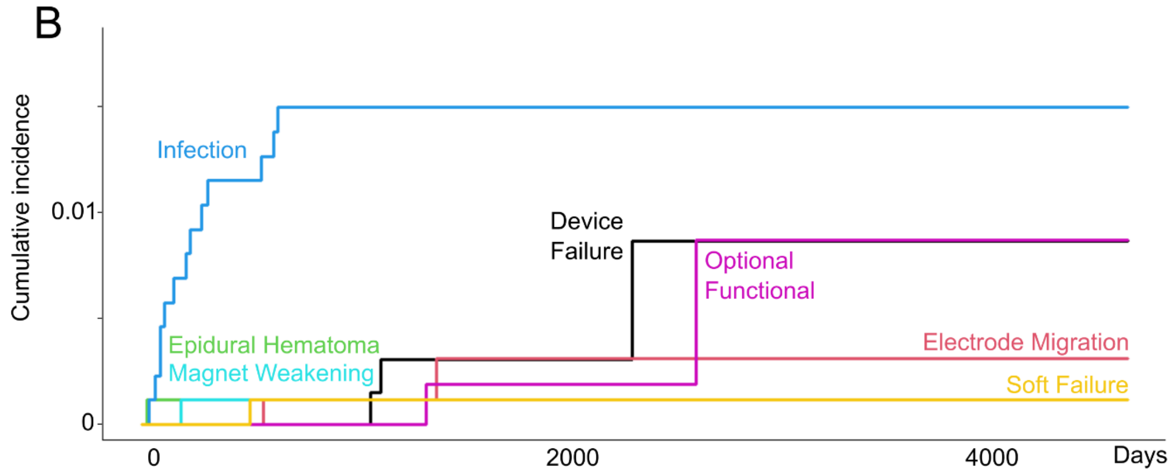

Supplement: Supplementary file 2 — Supplementary file2 (PDF 176 KB) [file 405_2026_10012_MOESM2_ESM.pdf]
